# Impact of LHRHa Therapy on Relationship Dynamics and Sexual Coercion in Premenopausal Breast Cancer Patients: A Multicenter Cross-Sectional Study

**DOI:** 10.3390/healthcare14010068

**Published:** 2025-12-26

**Authors:** Mustafa Ersoy, Canan Kaş

**Affiliations:** 1 Department of Internal Medicine, Faculty of Medicine, Kutahya Health Sciences University, Kutahya 43100, Turkey; 2 Department of Midwifery, Faculty of Health Sciences, Kastamonu University, Kastamonu 43100, Turkey; canankas@gmail.com

**Keywords:** breast cancer, luteinizing hormone-releasing hormone agonist, sexual coercion, tamoxifen, psychosocial oncology, supportive care

## Abstract

**Highlights:**

**What are the main findings?**

- Premenopausal breast cancer patients receiving LHRHa therapy showed significantly higher SCIRS scores across all domains compared with tamoxifen users.
- The Commitment Manipulation domain demonstrated the largest difference, indicating deeper relational strain associated with abrupt medical menopause.

**What are the implications of the main findings?**

- Findings highlight the need for routine psychosocial and sexual health screening in patients starting or receiving LHRHa therapy.
- Clinicians should consider age-sensitive supportive interventions, as younger women exhibited greater vulnerability to coercive relationship dynamics.

**Abstract:**

**Background/Objectives**: Luteinizing hormone-releasing hormone agonists (LHRHa) are widely used to induce ovarian suppression in premenopausal women with hormone receptor-positive breast cancer. Although effective, abrupt medical menopause may negatively affect sexual health and intimate partner interactions. Sexual coercion—ranging from manipulation to explicit pressure—remains an underrecognized psychosocial burden in oncology. This multicenter study aimed to evaluate the association between LHRHa therapy and sexual coercion, including relational dynamics measured through the Sexual Coercion in Intimate Relationships Scale (SCIRS). **Methods**: This cross-sectional study included 81 premenopausal breast cancer patients receiving endocrine therapy at three tertiary centers in Türkiye. Participants were categorized into tamoxifen monotherapy users (n = 39) and LHRHa users (n = 42). Sexual coercion was assessed using the validated Turkish SCIRS, which includes Resource Manipulation/Violence, Defection Threat, and Commitment Manipulation domains. Mann–Whitney U, Kruskal–Wallis, and ANCOVA analyses were performed, adjusting for age, treatment duration, surgery type, chemotherapy, radiotherapy, and education level. The study was ethically approved (2023-KAEK-148) and prospectively registered (NCT06840847). **Results**: LHRHa users demonstrated significantly higher SCIRS scores across all domains compared with non-users (RM/V: *p* = 0.039; DT: *p* = 0.001; CM: *p* < 0.001; Total: *p* = 0.004). ANCOVA confirmed LHRHa therapy as an independent predictor after adjusting for covariates (*p* = 0.001–0.006). The largest effect was observed in the Commitment Manipulation domain (partial η^2^ = 0.177). Younger patients (≤ 36 years) reported significantly greater coercion exposure across all domains (*p* = 0.018–0.042). **Conclusions**: LHRHa therapy is associated with increased sexual coercion and strained relational dynamics in premenopausal breast cancer patients, particularly among younger women. These findings emphasize the need for routine sexual health assessment, confidential psychosocial screening, and age-sensitive supportive interventions in endocrine therapy management.

## 1. Introduction

Breast cancer remains the most frequently diagnosed malignancy among women worldwide, with more than 2.3 million new cases reported in 2020 [1]. Approximately 80% of breast tumors are estrogen receptor-positive, making endocrine therapy a cornerstone of treatment [2]. In premenopausal patients, tamoxifen has long been the standard option, whereas ovarian function suppression with luteinizing hormone-releasing hormone agonists (LHRHa)—either alone or in combination with tamoxifen or aromatase inhibitors—has increasingly been adopted following the results of the SOFT and TEXT trials [3]. Although LHRHa therapy improves disease outcomes, it induces an abrupt, medically induced menopause, which is frequently associated with more severe and persistent symptoms than natural menopause [4,5].

Sexual dysfunction is among the most common and distressing survivorship issues in breast cancer patients [6]. Symptoms such as vaginal dryness, dyspareunia, reduced libido, diminished orgasmic capacity, and negative changes in body image can impair quality of life and place substantial strain on intimate relationships [7,8]. Recent studies from Europe, North America, and Latin America also highlight the complex relational impact of treatment-induced sexual dysfunction in breast cancer survivors. Large cohort studies from the United States and Canada have shown that endocrine therapy-related menopausal symptoms are associated with increased relational tension, reduced intimacy, and partner-driven pressure to maintain sexual activity [9,10]. Similarly, research from Brazil and Mexico has documented how sociocultural expectations and limited communication around sexuality may predispose women to coercive or guilt-based partner behaviors during survivorship [11,12,13]. These findings underscore the need to better understand how ovarian suppression may influence intimate partner dynamics across diverse cultural contexts.

While the physical aspects of sexual dysfunction have been extensively described, the psychosocial consequences—particularly those related to intimate partner behaviors—remain insufficiently explored [14]. Sexual coercion encompasses a spectrum of behaviors ranging from subtle manipulation to explicit pressure or threats regarding sexual activity [15] and is associated with impaired mental health, reduced relationship satisfaction, and negative survivorship outcomes [16]. These behaviors reflect broader relationship dynamics, including power imbalance, emotional manipulation, and partner control, which may be exacerbated when patients experience treatment-induced sexual difficulties.

Sociocultural factors strongly influence how women perceive and disclose sexual concerns [17]. In Türkiye, as in many conservative societies, discussions about sexuality may be stigmatized, leading women to underreport sexual dysfunction or coercive experiences [18]. Structured and validated instruments, such as the Sexual Coercion in Intimate Relationships Scale (SCIRS), provide a culturally appropriate and reliable means of assessing these sensitive issues [19].

Despite the widespread use of endocrine therapy in breast cancer management, little is known about how specific treatments—particularly ovarian suppression with LHRHa—may influence coercive partner behaviors and relationship dynamics. The theoretical rationale linking LHRHa therapy to sexual coercion lies in the profound physiological and psychological disruption caused by abrupt ovarian suppression [20]. Unlike the gradual transition of natural menopause, LHRHa induces a sudden and severe decline in estrogen, often resulting in marked hypoactive sexual desire and dyspareunia [21]. This creates a substantial ‘sexual desire discrepancy’ between the patient and her partner.

From a psychosocial perspective, this discrepancy may destabilize dyadic dynamics. Partners may misinterpret the patient’s biologically driven sexual withdrawal as emotional rejection [22]. In response, particularly within relationships shaped by traditional gender norms, partners may adopt coercive behaviors—ranging from guilt induction (‘Commitment Manipulation’) to threats of abandonment or infidelity (‘Defection Threat’)—as maladaptive attempts to restore intimacy or reassert relational control [23].

Based on this framework, we hypothesized that premenopausal women receiving LHRHa therapy would exhibit higher levels of sexual coercion and relational strain compared with those treated with tamoxifen monotherapy, independent of demographic and clinical variables. Therefore, this multicenter study aimed to compare experiences of sexual coercion between women treated with tamoxifen monotherapy and those receiving LHRHa therapy, using a validated assessment tool to provide evidence for improved psychosocial and supportive care in premenopausal breast cancer survivors.

## 2. Materials and Methods

### 2.1. Study Design and Setting

This multicenter, cross-sectional survey was conducted between December 2023 and July 2024 at three tertiary care institutions in Turkey: Kastamonu Education and Research Hospital, Kutahya Health Sciences University Evliya Celebi Education and Research Hospital, and Kutahya City Hospital. The study adhered to the Strengthening the Reporting of Observational Studies in Epidemiology (STROBE) guidelines for cross-sectional studies [24]. Ethical approval for this study was obtained from the Kastamonu University Clinical Research Ethics Committee (decision no. 2023-KAEK-148). Written informed consent was obtained from all participants in accordance with the principles of the Declaration of Helsinki. The study was prospectively registered at ClinicalTrials.gov (identifier: NCT06840847).

This study was purely observational in nature, without any random assignment or experimental procedures. Treatment groups reflected the endocrine therapy regimen that patients were already receiving in routine clinical practice.

### 2.2. Participants

Eligible participants were premenopausal women aged ≥18 years with a history of surgery for hormone receptor-positive breast cancer who were currently receiving endocrine therapy and were in an active intimate relationship. Exclusion criteria included metastatic disease, receipt of chemotherapy within the preceding three months, a history of psychiatric illness or neurocognitive impairment, and prior gynecological surgery that could independently affect sexual function.

The final sample (n = 81) reflects all eligible premenopausal women who met the inclusion criteria, were approached during the study period across the three centers, and voluntarily agreed to participate.

### 2.3. Treatment Groups

Participants were categorized into two groups: those receiving tamoxifen monotherapy (n = 39) and those undergoing LHRHa therapy (n = 42). The latter group included patients treated with monthly or three-monthly formulations of goserelin or leuprolide, administered either in combination with tamoxifen or with an aromatase inhibitor.

The age cutoff (≤36 years) was chosen based on subgroup findings from the SOFT and TEXT trials, which identify mid-30s as a clinically distinct premenopausal group with more severe ovarian suppression–related symptoms. This threshold also corresponded to the median age of our sample, enabling balanced group sizes for adjusted analyses.

### 2.4. Measures

Sexual coercion was assessed using the Turkish version of the SCIRS, a validated instrument that evaluates three domains of coercive partner behavior: Resource Manipulation/Violence (RM/V), Defection Threat (DT), and Commitment Manipulation (CM). Each item is rated on a six-point Likert scale, with higher scores indicating greater exposure to coercion.

All interviews were conducted individually in a confidential setting by trained female clinicians with at least two years of experience in breast cancer survivorship care. Interviewers received standardized training on SCIRS administration to minimize interviewer-related variability. When needed, participants were offered referrals for psychological counseling. Missing data were minimal and handled using complete-case analysis, and no systematic interviewer effects were identified during data quality checks.

The SCIRS consisted of 39 items divided into three subscales consistent with its original structure: CM (20 items), DT (12 items), and RM/V (7 items). Internal consistency in the present sample was excellent, with Cronbach’s α values of 0.966 for the total scale, 0.952 for CM, 0.912 for DT, and 0.831 for RM/V.

### 2.5. Statistical Analysis

Descriptive statistics were calculated for all demographic and clinical variables. Group differences in categorical variables were analyzed using the chi-square test or Fisher’s exact test, as appropriate. Between-group comparisons of SCIRS scores were performed using the Mann–Whitney U test for binary comparisons (e.g., LHRHa users vs. non-users; ≤36 vs. >36 years) and the Kruskal–Wallis test for comparisons across more than two groups (e.g., educational level).

Adjusted univariate ANCOVA models were constructed for each SCIRS domain and the total score, with LHRHa use entered as the fixed factor. To minimize the risk of overfitting given the sample size (n = 81), the number of covariates in the adjusted models was strictly limited. Only variables that demonstrated statistically significant associations in preliminary bivariate analyses or were identified as essential confounders in prior literature (e.g., age group) were retained. All ANCOVA assumptions were examined prior to model interpretation. Homogeneity of regression slopes was assessed by testing the interaction between each covariate and the treatment group; no significant violations were detected. Variance equality was evaluated using both Levene’s and Brown-Forsythe tests. When variance heterogeneity was present, Welch-adjusted F statistics and robust standard errors were applied. Residual distributions were evaluated via Q-Q plots and Shapiro–Wilk tests, confirming acceptable normality. Effect sizes were expressed as r values for Mann–Whitney U tests and partial η^2^ for ANCOVA, using thresholds of 0.01, 0.06, and 0.14 to indicate small, medium, and large effects, respectively. Statistical significance was set at *p* < 0.05 (two-sided). In addition, Mann–Whitney U tests were performed as non-parametric sensitivity analyses for each SCIRS domain to ensure consistency of findings given the ordinal nature of the scale.

## 3. Results

### 3.1. Demographic and Clinical Characteristics

A total of 81 breast cancer patients were analyzed, including 42 patients receiving LHRHa therapy and 39 patients treated with tamoxifen monotherapy. Baseline demographic and clinical characteristics are summarized in Table 1. Both groups were largely comparable in terms of education, treatment duration, surgery type, receipt of chemotherapy, and receipt of radiotherapy. The only significant difference was age distribution, with younger patients being more prevalent in the LHRHa group (*p* = 0.036).

### 3.2. Internal Reliability of the SCIRS

Internal reliability analyses demonstrated excellent internal consistency for the SCIRS total score (Cronbach’s α = 0.966). Subscale reliability was also high: Commitment Manipulation (α = 0.952), Defection Threat (α = 0.912), and Resource Manipulation/Violence (α = 0.831). These findings indicate that the scale performed reliably within this breast cancer cohort.

### 3.3. Age and Educational Level

When stratified by age, women aged ≤36 years reported significantly higher partner coercion scores across all SCIRS domains compared with those older than 36 years (RM/V: *p* = 0.042; DT: *p* = 0.018; CM: *p* = 0.027; Total: *p* = 0.024; Table 2).

In contrast, educational level did not significantly influence SCIRS scores in any domain (all *p* > 0.05; Table 3).

### 3.4. LHRHa Use and Coercion Outcomes

Mann–Whitney U tests demonstrated significantly higher coercion scores among LHRHa users compared with non-users across all SCIRS domains (RM/V: *p* = 0.039; DT: *p* = 0.001; CM: *p* < 0.001; Total: *p* = 0.004). Effect sizes ranged from small–medium to medium–large, with the greatest difference observed in the Commitment Manipulation domain (r = 0.41; Table 4).

### 3.5. Adjusted Analyses

**Figure 1:**
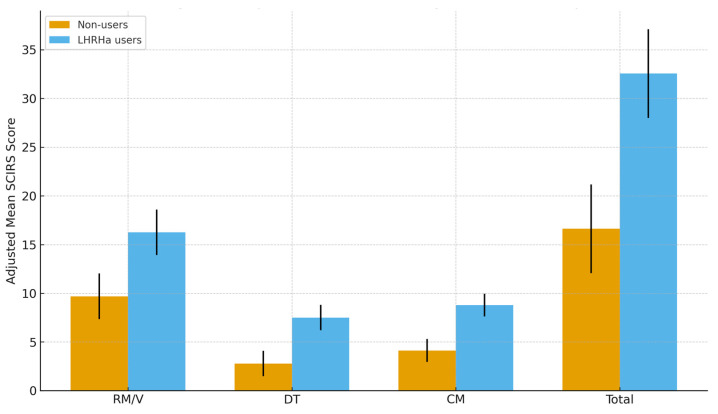
Adjusted mean SCIRS scores across treatment groups. Bar plots illustrate the mean scores for non-users and LHRHa users in each domain of the Sexual Coercion in Intimate Relationships Scale (SCIRS): Resource Manipulation/Violence (RM/V), Defection Threat (DT), Commitment Manipulation (CM), and Total score. Error bars represent standard errors. *p* values are derived from ANCOVA models adjusted for age group, treatment duration, surgery type, receipt of chemotherapy, receipt of radiotherapy, and education level.

To account for potential confounders, analysis of covariance (ANCOVA) models were applied with LHRHa use as the fixed factor. Consistent with the strict covariate selection approach described in the Methods Section 2, only theoretically essential or bivariately associated variables (age group, treatment duration, surgery type, receipt of chemotherapy, receipt of radiotherapy, and education level) were retained in the adjusted models. All ANCOVA assumptions were examined prior to interpretation, including homogeneity of regression slopes, normality of residuals, and variance equality. Welch-adjusted F statistics and robust standard errors were applied when variance heterogeneity was detected. As shown in Table 5 and Figure 1, LHRHa therapy remained significantly associated with higher coercion scores across all domains (RM/V: *p* = 0.006; DT: *p* = 0.001; CM: *p* < 0.001; Total: *p* = 0.001). Partial η^2^ values ranged from 0.098 to 0.177, indicating medium-to-large effect sizes and providing additional support for the robustness of these observed associations despite the modest sample size. These findings should be interpreted as associative rather than causal, given the cross-sectional design and the ordinal nature of the SCIRS scale.

## 4. Discussion

This multicenter study examined the association between endocrine therapy type and intimate partner sexual coercion among premenopausal women with breast cancer. The findings showed that women receiving LHRHa therapy reported significantly higher SCIRS scores across all domains compared with those treated with tamoxifen monotherapy. These differences remained significant after adjustment for demographic and treatment-related factors, indicating that ovarian suppression itself—rather than age, treatment duration, surgery type, or adjuvant therapies—was an independent contributor to increased coercive relational dynamics.

The relationship between endocrine therapy and sexual health has been well established in the oncology literature. LHRHa induces abrupt medical menopause, which is associated with more severe vasomotor, emotional, and urogenital symptoms compared to natural menopause [4,5]. Prior studies have highlighted that sexual dysfunction—including reduced libido, dyspareunia, vaginal dryness, diminished orgasmic capacity, and negative changes in body image—is highly prevalent among breast cancer survivors and often more intense in those undergoing ovarian suppression [6,7]. Sexual difficulties significantly affect psychological well-being and have been reported to contribute to marital strain, reduced emotional intimacy, and overall deterioration in relationship quality [8,25]. Our findings build on this evidence by demonstrating that these treatment-related changes may extend beyond sexual discomfort and influence broader relationship dynamics, including manipulation, emotional pressure, and threats of relationship dissolution.

The most pronounced group difference in our study was observed in the Commitment Manipulation and Defection Threat domains, which reflect relational power imbalance and emotional coercion. These findings suggest that when women experience abrupt sexual and emotional changes due to LHRHa, partners may respond with pressure, dissatisfaction, or attempts to assert relational control. Such patterns are consistent with previous research showing that sexual dysfunction can heighten relational conflict and contribute to maladaptive partner behaviors [26]. Importantly, these behaviors may occur even in relationships without formal physical violence, highlighting the necessity of evaluating subtle but harmful relational patterns.

Age emerged as another key factor, with younger women (≤36 years) reporting significantly higher coercion across all SCIRS domains. This observation aligns with prior studies showing that younger breast cancer survivors report more severe sexual distress, higher levels of marital conflict, and an increased risk of separation or divorce compared with older women [27,28]. Younger women are more likely to be in sexually active relationships, raising young families, or navigating earlier stages of marital development, which may intensify vulnerability to relational strain during cancer treatment. Additionally, abrupt treatment-induced menopause may challenge expectations related to femininity, sexuality, and reproductive potential, leading to heightened concerns about fertility loss and disruptions in identity. These mechanisms may amplify emotional distress and relational tension, thereby contributing to the higher coercion scores observed in this group.

Educational level, however, did not significantly influence coercion outcomes. This may indicate that cancer-related sexual and relational difficulties transcend traditional sociodemographic boundaries. Previous studies have similarly shown that treatment-induced changes in sexual health and partner communication affect women across all educational and socioeconomic backgrounds [29,30]. These results emphasize the importance of clinical, rather than demographic, variables in shaping the psychosocial experience of breast cancer survivors.

Sociocultural context also plays a critical role in interpreting our findings. In conservative societies, including Türkiye, discussions about sexuality often carry stigma, which may discourage women from openly expressing sexual concerns or seeking help [17,18]. Such cultural norms may contribute to underrecognition of sexual coercion and relational distress in routine oncology care. The use of a validated, culturally appropriate instrument such as the SCIRS was therefore crucial for accurately assessing these sensitive issues [19]. Our results underscore the need for structured, confidential, and proactive screening strategies to identify patients experiencing relational strain or coercion, particularly those undergoing ovarian suppression. Moreover, in sociocultural settings where fertility and reproductive capacity are closely tied to femininity, identity, and marital expectations, abrupt treatment-induced menopause may heighten emotional distress and alter partner perceptions [31]. The awareness or fear of reduced fertility can influence both women’s sense of self and their partners’ relational behaviors, potentially intensifying vulnerability to coercive dynamics—especially among younger patients [32]. Our findings are consistent with international evidence showing that treatment-related sexual dysfunction can reshape intimate partner dynamics. Studies from Europe, North America, and Latin America have similarly reported increases in relational tension, emotional pressure, and coercive partner responses during survivorship, suggesting that the psychosocial impact of ovarian suppression may extend across diverse cultural settings [9,10,11,12,13].

Clinically, these findings highlight the need for comprehensive psychosocial and sexual health assessment in premenopausal women receiving LHRHa therapy. Oncology providers should openly discuss potential sexual and relational consequences during treatment planning and ensure confidential environments that facilitate disclosure of sensitive concerns. Integrating psycho-oncology services, sexual counseling, and supportive interventions may reduce relational stress and improve survivorship outcomes. Partner education about treatment-related changes can foster empathy and prevent maladaptive reactions. Because coercive dynamics arise within the couple, incorporating partner-focused or dyadic counseling may further enhance communication and reduce relational tension. Future survivorship programs should therefore consider structured psychological education and partner-inclusive support.

This study has several limitations. First, its cross-sectional observational design precludes causal inference and allows only associative interpretations, particularly regarding the complex relationships between endocrine therapy, psychological responses, and coercive partner behaviors. Additionally, the relational mechanisms discussed in this study should be interpreted as theoretical rather than causal, as this design cannot determine whether treatment-induced menopausal symptoms directly lead to coercive partner behaviors. Second, the reliance on self-reported data may introduce recall error and social desirability bias, especially in conservative sociocultural contexts where discussions about sexuality and relational difficulties may be stigmatized. Although all assessments were conducted privately by trained female clinicians, underreporting of coercive experiences remains possible and may have resulted in conservative effect estimates.

Third, because SCIRS scores originate from ordinal Likert-type items, the use of parametric ANCOVA models should be interpreted with caution. The modest sample size increases the risk of overfitting, particularly when multiple covariates are included. Although all ANCOVA assumptions—including normality of residuals, variance equality, and homogeneity of regression slopes—were examined and satisfied, variance heterogeneity was detected in some models and addressed using Welch-adjusted F statistics and robust standard errors. Therefore, the adjusted estimates should be interpreted cautiously.

Fourth, the study did not incorporate broader psychosocial constructs—such as anxiety, depression, body image, fertility concerns, self-esteem, marital satisfaction, or pre-existing relationship conflict—which may meaningfully influence both women’s vulnerability to coercive dynamics and their adjustment to treatment-induced menopause. Although counseling regarding ovarian suppression is routinely offered, the extent to which women understood the sexual and psychosocial consequences of treatment was not formally assessed; this unmeasured factor may influence functioning and perceived coercion. Additionally, socioeconomic status, motherhood status, and relationship duration or partner history were not collected, and these unmeasured factors may further contribute to relational vulnerability.

Fifth, interviewer influence cannot be completely excluded despite standardized SCIRS administration training. Moreover, partner-level factors—such as partners’ beliefs, expectations, coping styles, or psychological distress—were not assessed, though they may shape relational dynamics following endocrine therapy.

Finally, the study population was recruited from a single geographic region, which may limit generalizability. Future research should employ longitudinal designs with repeated assessments before and after endocrine therapy initiation and incorporate comprehensive psychosocial and partner-level variables to better characterize temporal changes and causal pathways in coercive relational dynamics.

In summary, this study demonstrates that LHRHa therapy is associated with significantly higher levels of intimate partner sexual coercion and strained relational dynamics in premenopausal breast cancer patients, particularly among younger women. These findings underscore the importance of integrating sexual health evaluation, psychosocial screening, and individualized supportive care into the management of endocrine therapy, with special attention to the relational and emotional needs of this vulnerable patient population.

## 5. Conclusions

In conclusion, premenopausal women receiving LHRHa therapy demonstrated significantly higher levels of intimate partner sexual coercion compared with those treated with tamoxifen alone; however, these findings are associative rather than causal. The results underscore the need for clinicians to recognize the relational and psychosocial challenges that may accompany abrupt ovarian suppression, particularly in sociocultural settings where open communication about sexuality is limited. Routine assessment of sexual well-being, relational strain, and partner dynamics should be incorporated into survivorship care, with special attention to younger women, who appear more vulnerable to coercive relational patterns. Future longitudinal and multicenter studies with larger and more diverse cohorts are needed to clarify temporal trajectories, identify high-risk subgroups, and better elucidate the mechanisms through which endocrine therapy influences intimate partner relationships.

## Figures and Tables

**Table 1:** Demographic and Clinical Characteristics of Patients by LHRHa Use.

Characteristic	LHRHa Non-Users (n = 39)	LHRHa Users (n = 42)	Total (n = 81)	*p* Value
Age group				**0.036**
≤36 years	16 (41.0%)	27 (64.3%)	43 (53.1%)	
>36 years	23 (59.0%)	15 (35.7%)	38 (46.9%)	
Education				0.276
Elementary school	4 (10.3%)	6 (14.3%)	10 (12.3%)	
Middle school	11 (28.2%)	9 (21.4%)	20 (24.7%)	
High school	10 (25.6%)	18 (42.9%)	28 (34.6%)	
University	14 (35.9%)	9 (21.4%)	23 (28.4%)	
Treatment duration				0.637
≤2 years	25 (64.1%)	29 (69.0%)	54 (66.7%)	
>2 years	14 (35.9%)	13 (31.0%)	27 (33.3%)	
Surgery type				0.448
Mastectomy	19 (48.7%)	24 (57.1%)	43 (53.1%)	
Breast-conserving surgery	20 (51.3%)	18 (42.9%)	38 (46.9%)	
Received chemotherapy				0.986
Yes	25 (64.1%)	27 (64.3%)	52 (64.2%)	
No	14 (35.9%)	15 (35.7%)	29 (35.8%)	
Received radiotherapy				0.586
Yes	19 (48.7%)	23 (54.8%)	42 (51.9%)	
No	20 (51.3%)	19 (45.2%)	39 (48.1%)	

Note. Values are presented as n (%). *p* values were calculated using the Chi-square test or Fisher’s exact test when expected cell counts were <5. Bold *p*-values indicate statistical significance (*p* < 0.05). LHRHa = Luteinizing Hormone-Releasing Hormone agonist.

**Table 2:** Partner Coercion Exposure in Breast Cancer Patients by Age Group.

SCIRS Domain	≤36 Years (n = 43) Mean Rank	>36 Years (n = 38) Mean Rank	U	Z	*p* Value
RM/V	45.99	35.36	602.5	−2.033	**0.042**
DT	46.69	34.57	572.5	−2.366	**0.018**
CM	46.42	34.87	584.0	−2.215	**0.027**
Total	46.53	34.74	579.0	−2.254	**0.024**

Note. Values are mean ranks from the Mann–Whitney U test (UDMAT-Winnie). U, Z, and *p* values are reported for each comparison. Bold *p*-values indicate statistical significance (*p* < 0.05). RM/V = Resource Manipulation/Violence; DT = Defection Threat; CM = Commitment Manipulation; SCIRS = Sexual Coercion in Intimate Relationships Scale.

**Table 3:** Partner Coercion Exposure in Breast Cancer Patients by Educational Level.

SCIRS Domain	Elementary (n = 10)	Middle (n = 20)	High (n = 28)	University (n = 23)	χ^2^ (df)	*p* Value
RM/V	41.85	42.48	43.46	36.35	1.302 (3)	0.729
DT	46.00	40.03	45.21	34.54	3.259 (3)	0.353
CM	44.65	40.63	44.73	35.20	2.372 (3)	0.499
Total	42.75	41.83	44.43	35.35	2.006 (3)	0.571

Note. Values represent mean ranks from the Kruskal–Wallis test. χ^2^ statistics and corresponding *p*-values are provided for each domain. *p* < 0.05 was considered statistically significant. RM/V = Resource Manipulation/Violence; DT = Defection Threat; CM = Commitment Manipulation; SCIRS = Sexual Coercion in Intimate Relationships Scale.

**Table 4:** Partner Coercion Exposure in Breast Cancer Patients by LHRHa Use.

SCIRS Domain	Non-Users (n = 39) Mean Rank	Users (n = 42) Mean Rank	U	Z	*p* Value	Effect Size (r)
RM/V	35.40	46.20	600.5	−2.068	**0.039**	0.23 (small–medium)
DT	32.09	49.27	471.5	−3.359	**0.001**	0.37 (medium)
CM	30.99	50.30	428.5	−3.708	**0.000**	0.41 (medium–large)
Total	33.10	48.33	511.0	−2.914	**0.004**	0.32 (medium)

Note. Non-parametric comparisons were performed using the Mann–Whitney U test (UDMAT-Winnie). *r* effect sizes were calculated as *r* = *Z/*√*N*. Bold *p*-values indicate statistical significance (*p* < 0.05). RM/V = Resource Manipulation/Violence; DT = Defection Threat; CM = Commitment Manipulation; SCIRS = Sexual Coercion in Intimate Relationships Scale.

**Table 5:** Adjusted Effects of LHRHa Use on SCIRS Scores (ANCOVA).

SCIRS Domain	Adjusted Mean (Users)	Adjusted Mean (Non-Users)	F (1, 73)	*p* Value	Partial η^2^	Interpretation
RM/V (KYZ)	16.26	9.69	7.890	**0.006**	0.098	Medium effect
DT (AT)	7.50	2.79	13.036	**0.001**	0.152	Medium–large effect
CM (BM)	8.79	4.13	15.744	**0.000**	0.177	Large effect
SCIRS Total	32.55	16.62	12.231	**0.001**	0.144	Medium–large effect

Note. Adjusted means are reported from ANCOVA models controlling for age group, treatment duration, surgery type, receipt of chemotherapy, receipt of radiotherapy, and education level. Partial η^2^ values represent effect sizes (small ≈ 0.01, medium ≈ 0.06, large ≈ 0.14). Bold *p* values indicate statistical significance at *p* < 0.05. RM/V = Resource Manipulation/Violence; DT = Defection Threat; CM = Commitment Manipulation; SCIRS = Sexual Coercion in Intimate Relationships Scale.

## Data Availability

The datasets generated and analyzed during the current study are available from the corresponding author upon reasonable request.

## Abbreviations

LHRHa	Luteinizing Hormone-Releasing Hormone agonist
SCIRS	Sexual Coercion in Intimate Relationships Scale
RM/V	Resource Manipulation/Violence
DT	Defection Threat
CM	Commitment Manipulation
ANCOVA	Analysis of Covariance
